# Initial Conditioning of Used Cigarette Filters for Their Recycling as Acoustical Absorber Materials

**DOI:** 10.3390/ma14154161

**Published:** 2021-07-27

**Authors:** Valentín Gómez Escobar, Celia Moreno González, María José Arévalo Caballero, Ana Mᵃ Gata Jaramillo

**Affiliations:** 1 Departamento de Física Aplicada, Escuela Politécnica, Universidad de Extremadura, Avda. de la Universidad s/n, 10003 Cáceres, Spain; celiamg@unex.es; 2 Departamento de Química Orgánica e Inorgánica, Escuela Politécnica, Universidad de Extremadura, Avda. de la Universidad s/n, 10003 Cáceres, Spain; arevalo@unex.es (M.J.A.C.); anamariagj@unex.es (A.M.G.J.)

**Keywords:** sound absorber, cigarette butts, sustainable material, recycling, chemical cleaning

## Abstract

Used cigarette butts represent a major and problematic form of waste, due to their abundance, toxicity, and durability. Moreover, the few proposals for their recycling are clearly insufficient, and new ones are welcome. For a new proposal regarding the reuse of used cigarette butts as acoustical absorbers in building construction, previous conditioning of the used butts is performed. This conditioning includes the elimination of moisture and toxic products accumulated in the filter of the cigarettes. Thus, in this work, the moisture content effect in acoustical absorption was analyzed, and a proposal for elimination is made. Moreover, a chemical cleaning procedure is proposed, and its influence on the acoustical behavior of the samples was also analyzed.

## 1. Introduction

Cigarette butts (mainly the filters of the millions of cigarettes consumed) are among the most problematic types of global waste. The importance of the problem derived from this waste can be determined by the four components described below.

Firstly, as smoking is commonly practiced all around the world, trillions of cigarettes are consumed each year on a global scale [1,2]. This amount is even expected to increase by more than 50% by 2025 due both to the increase in the world population and in tobacco production [3]. Almost all these smoked cigarettes incorporate a filter as a consequence of studies carried out in the middle of the twentieth century demonstrating that its use reduces the risk of lung cancer in smokers. Thus, trillions of used filter cigarettes are generated annually.

Secondly, unfortunately, cigarette butts are commonly thrown on the ground [4]. Thus, cigarette butts can be found in almost all environments throughout the world. In fact, cigarette butts are usually the major element in terms of quantity in garbage and even in terms of weight in some cases [5,6,7]. This second component is even more relevant when linking it with the two components presented below.

Thirdly, butts thrown on the ground are carried by rain or river water to coastal areas, increasing their environmental impact. This environmental impact of cigarette butts is related to both their chemical composition [8] and the difficulty of degradation of cellulose acetate. Thus, some 130 chemical substances present in cigarette butts have been described, chemicals that can be easily leached into water, especially toxic heavy metals, nicotine, and additives [9,10], thereby making these waters toxic to different organisms [8,11,12].

Fourthly, almost all cigarette filters are made of cellulose acetate, a polymer that undergoes a very slow biological degradation and takes several months to photodegrade (only partial photodegradation occurs since the polymer chains are cleaved into smaller pieces) [13]. Therefore, this waste can remain in different environments for a long time.

From the above components, it can be concluded that used cigarette butts constitute a serious environmental and public health problem, as has been stated in a recent study by the World Health Organization [14].

These four components are associated with a fifth, which is the fact that despite the described problem of this waste, there are few initiatives for its recycling. Some authors have developed several proposals to recycle this residue (for example, in the manufacture of supercapacitors [15], as chemical corrosion inhibitors [16], and as a component of bricks [17]). Most of the initiatives proposed in recent years are compiled in two recently published papers [18,19]. The existing proposals, however, are scarce and very limited because of the number of cigarette butts that they require, and they are clearly insufficient, considering the continuous generation of this waste.

As a new proposal for recycling this waste, over the past few years our research group has been working on the elaboration of an acoustical absorber material obtained from used cigarette butts. In these studies [20,21,22,23], absorption of the prepared samples (some of them desegregating the cigarette butt filters, but mainly manually inserting them) was fairly satisfactory, being comparable to or even better than those of some other materials conventionally used for sound absorption.

In these previous studies, however, some questions were not solved and are thus addresses in the present work. Therefore, this study examines the case of previous conditioning of samples and identifies the adequate chemical treatment in order to perform previous cleaning of filters.

Both issues (previous conditioning of samples and cleaning chemical treatment) are relevant when first considering the fact that the start samples (used cigarette butts) came from different places and that their moisture contents can differ. As such, we are interested in determining the influence of this moisture content on the samples’ acoustical properties, as well as establishing a standard procedure for their elimination. Moreover, the toxic character of this waste (due to the previously mentioned accumulation of toxic residues during the combustion) has made it necessary to develop a proposal of chemical cleaning of used cigarette butts.

Regarding this chemical cleaning, only a few of proposals consider the separation of the butts and the entrapped chemicals in them. For instance, cellulose acetate has been extracted and purified by soaking cigarette butts in distilled water and in ethanol to construct a cellulose-based membrane separator for a high-performance lithium-ion battery [24]; to prepare highly porous carbons as CO_2_ super absorbents [25]; or to obtain cellulose [26]. Further research is necessary to analyze the degree of chemical cleaning and to study possible alternatives to this cleaning.

Thus, the aims of this work are (1) to analyze the influence of moisture content on acoustical absorption and the procedure for moisture elimination, and (2) to propose an adequate chemical treatment process in order to carry out previous cleaning of filters and to characterize the influence of this chemical cleaning on the acoustical properties of the samples.

## 2. Materials and Methods

### 2.1. Preparation of Samples

Smoked cigarette butts from different brands were picked from ashtrays or from the ground in buildings from the Campus of the University of Extremadura and surroundings. They formed a very heterogeneous mixture; each butt had its original tobacco and blend of additives and different amounts of remaining unburnt tobacco, as well as other possible contaminants, such as lipstick and saliva residue. The remaining non-smoked tobacco and the external wrapping paper were manually separated, and only the cigarette butt filter were taken.

The remaining cigarette butt filters were nonhomogeneous. The length, diameter, presence of burnt regions, squashing degree, humidity, etc. differed for each butt. The most common lengths of filters were 12, 15, 20, and 26 mm, while the most common diameters were 6 and 8 mm.

**Figure 1:**
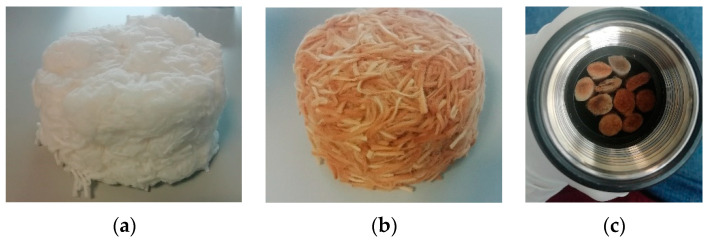
Example pictures of some of the prepared samples: (**a**) sample from disaggregated non-used cigarette butts; (**b**) sample from disaggregated used cigarette butts; (**c**) sample from used cigarette butts before the cleaning treatment.

Two different samples were used in the present study. For the chemical cleaning study, used cigarette butt filters (only without external wrapping paper, as previously mentioned) were used. However, in the analysis of the influence of moisture content and the drying conditions, in order to acquire a structure similar to that of the commercial acoustical products that are also included in the study, cigarette butts were prepared, disaggregating the filters and making a fiber mass. Figure 1 shows some pictures of the prepared samples.

### 2.2. Reagents and Solutions for Cleaning Used Cigarette Filters

Absolute ethanol, sulfuric acid (95–97% pure), and sodium chloride were purchased from Scharlab S. L. (Sentmenat, Spain). Ultrapure water was produced by an automatic water distillation apparatus (Barnstend Nanopure, Thermo Scientific, Waltham, MA, USA).

Aqueous solutions, 5% NaCl (*w*/*v*) and 0.02% H_2_SO_4_ (*w*/*v*), were prepared by adding the solid or the dilute solute to ultrapure water.

### 2.3. Cleaning Used Cigarette Filters

Samples of 15 butt filters (without the external the wrapping paper, as described previously) were extracted sequentially under stirring at room temperature with 125 mL of a 0.02% (*w*/*v*) H_2_SO_4_ aqueous solution (five times), with 125 mL of a 5% (*w*/*v*) NaCl aqueous solution (four times), and with 100 mL of ethanol (six times). Each extraction was carried out for 1 h. Cleaned butts were dried in an oven at 80 °C for 48 h. Then, they were characterized by nuclear magnetic resonance. ^1^H-NMR spectra were recorded on a Bruker Avance 500 MHz spectrometer (Billerica, MA, USA), using CDCl_3_ as solvent. The aqueous leachates were analyzed for heavy metals using an Agilent 7900 ICP mass spectrometer (Santa Clara, CA, USA).

### 2.4. Instrumentation for Acoustic Absorption Determination

**Figure 2:**
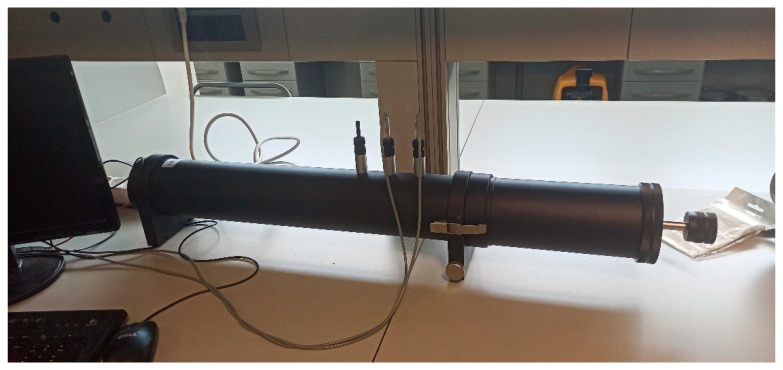
Impedance tube disposition used for the measurements.

Measurements of the absorption coefficient of the different samples were conducted using the Brüel & Kjær Impedance Tube Kit (Type 4206, Copenhagen, Denmark), equipped with two one-quarter-inch condenser microphones (Type 4187) (Figure 2). The signals were analyzed using a portable Brüel & Kjær PULSE System, with four input data channels (Type 3560-C). Two sample holders, with diameters of 29 mm (with a validity in the frequency range of 500 Hz to 6.4 kHz) and 100 mm (with a validity in the frequency range of 50 Hz to 1600 kHz) were used.

The determination of the sound absorption coefficient of different samples was carried out using an impedance tube following the two-microphone transfer function method described in the ISO 10534-2 standard [27].

**Figure 3:**
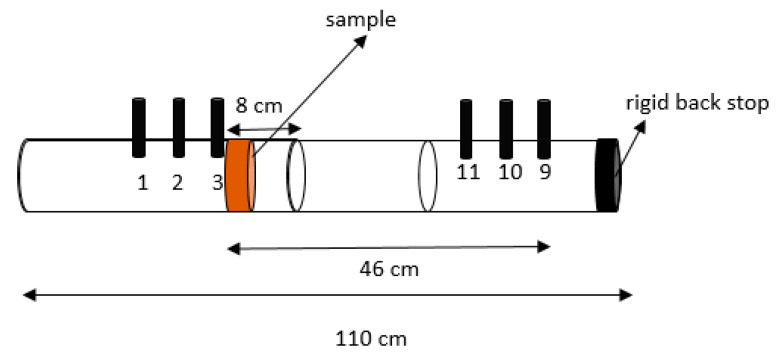
Scheme of the sample configuration for airflow resistivity determinations.

The impedance tube was also used for the determination of the airflow resistivity of some of the samples. This property is related to the resistance of the air to penetrate a porous material and, thus, is related to the absorption capacity of the material, as in porous material, a major part of the absorption is produced inside it. The units of this property are Pa·s/m^2^ or Rayls/m. For the determination of airflow resistivity, the Ingard and Dear method was used [28], with a configuration of sample inside the tube as is shown in Figure 3.

## 3. Results and Discussion

### 3.1. Previous Conditioning of Used Cigarette Butts Filters

As previously mentioned, the cigarette butt filters came from ashtrays or directly from the ground. Thus, their initial statuses differed; for instance, their humidity degrees were considerably different. As such, to homogenize the initial humidity degree of the used filters, they were dried.

In order to establish adequate drying conditions, six different samples (two made with used butt filters—samples UBF1 and UBF2; two made with non-used cigarette butts—samples NUBF1 and NUBF2; and two made with commercial absorption products—Mineral Wool 1 and Mineral Wool 2) were prepared. Firstly, they were dried for 48 h at 80 °C. Then, they were placed in a saturated environment for 50 days. The samples were weighted and acoustically characterized during the process.

Variation in weight of the prepared samples is presented in Table 1. As it can be seen, during the 50 days, all of their weights increased by around 13–21% due to the adsorption of water.

**Figure 4:**
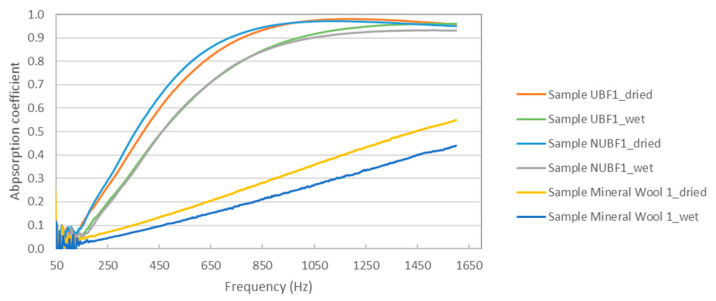
Variation in the absorption coefficient with the degree of humidity in the analyzed samples.

Is important to note that the change in weight of the samples produce a variation in their absorption, as can be seen in Figure 4 for three of the samples analyzed.

**Figure 5:**
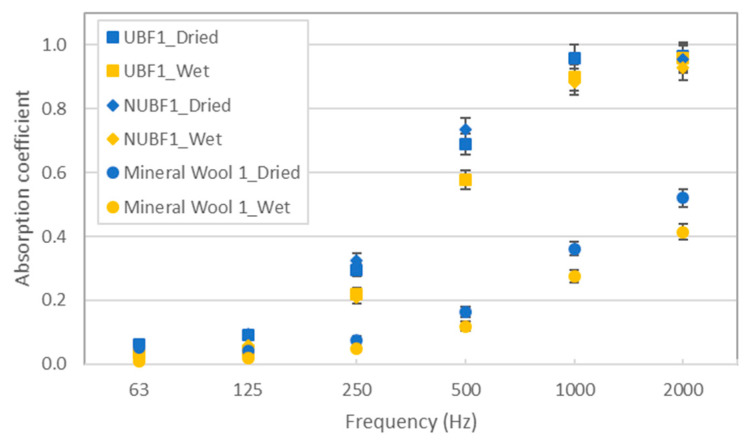
Variation in absorption coefficient with the degree of humidity in the analyzed samples (octave bands).

As can be seen in Figure 4, there is a clear reduction in the absorption capacity of the samples when they are saturated. Another way to quantify this reduction is by calculating the octave band coefficient absorption, which is represented in Figure 5. The average values of the absorption coefficient were 0.42 and 0.35 for the sample UBF1 (dried and wet, respectively); 0.43 and 0.35 for the sample NUBF1 (dried and wet, respectively); and 0.14 and 0.09 for the sample Mineral Wool 1 (dried and wet, respectively).

**Figure 6:**
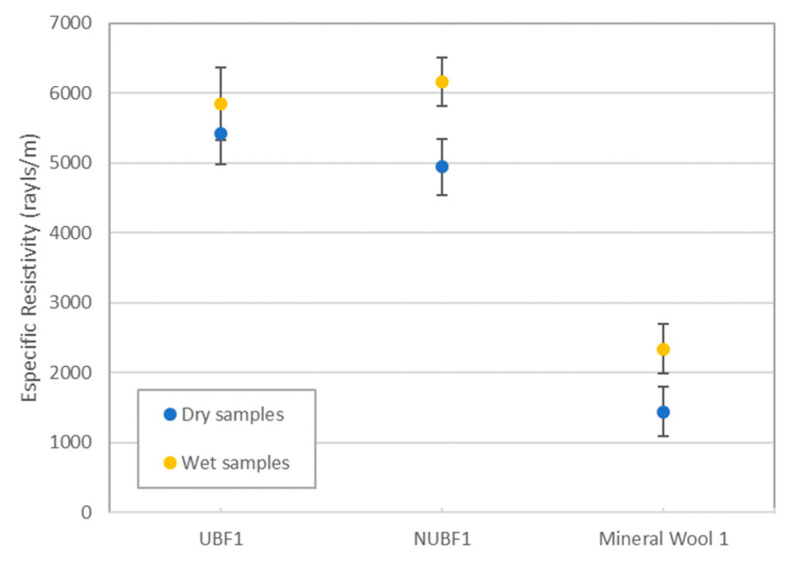
Variation of airflow resistivity of the samples shown in Figure 4.

This observed reduction in the absorption coefficient of the samples with moisture is probably due to the increase in the difficulty of the penetration of waves inside the material due the increase of the size of the fibers with moisture. This hypothesis is confirmed by the measured values of the airflow resistivity of the samples. As can be observed in Figure 6, there is an increase in the airflow resistivity values in the wet samples with respect to those of the dried ones, indicating increased difficulty of air to penetrate the materials and, thus, coherence with the reduction in the absorption coefficient values observed in Figure 4 and Figure 5.

In the second step, the wet samples were dried, three of them at 105 °C as indicated by the ISO 585 standard [29] and the other three at 80 °C. Variations in the weight of these samples, in percentage, are presented in Table 2.

As can be seen in Table 2, almost all the moisture content is eliminated, both for drying at 105 °C and at 80 °C, in the first three hours. Only a residual fraction (lower than 0.8% in the highest sample) is eliminated after the first three hours.

Thus, according to the results shown in Table 2, a drying period of three hours is sufficient (for drying at both 105 °C and 80 °C) in order to eliminate the moisture content of used cigarette butts from the presented samples.

### 3.2. Chemical Cleaning of the Used Cigarette Butts Filters

As previously mentioned, used cigarette butt filters have a number of toxic compounds inside that are generated during smoking. Moreover, their color and their smell are generally deemed to be unpleasant. Thus, a second use of this waste must be associated with previous cleaning and even an improvement to its image.

In order to identify an adequate procedure for the cleaning of the samples, several proofs in an attempt to use diluted reactants in order to reduce the posterior problem associated with the treatment of used reactive solutions were carried out. Furthermore, it was important that the used cigarette butts maintained the good acoustical behavior observed for the non-cleaned samples.

After several attempts, a good balance was found for a cleaning procedure based on sequential solid–liquid extractions with 0.02% H_2_SO_4_ (*w*/*v*), 5% NaCl (*w*/*v*), and absolute ethanol [30]. Sulfuric acid and sodium chloride aqueous solutions were used to extract heavy and trace metals that can be found in cigarette butts. Table 3 shows the concentration of different metals in the aqueous extracts. Either sulfuric acid or sodium chloride solutions allow the extraction of metals; the former appears to perform better for the extraction of Al, while NaCl is better at eliminating Pb, Mn, Sr, and Ba. Copper and cadmium were not found in the extracts.

**Figure 7:**
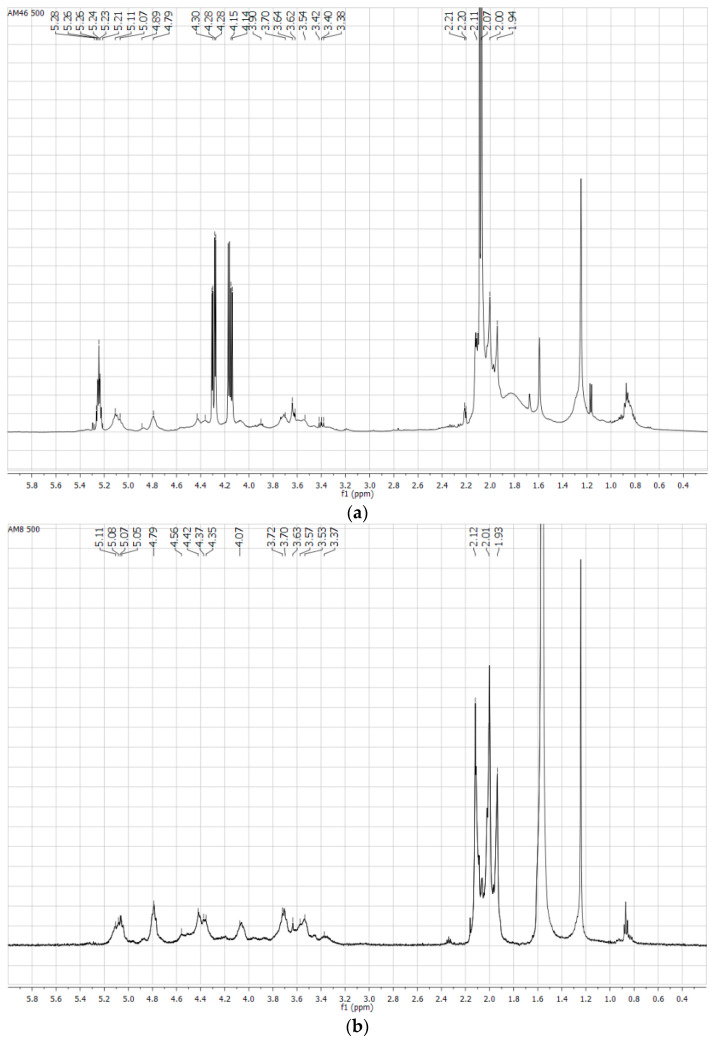
^1^H-NMR spectrum of butts before (**a**) and after (**b**) being submitted to the cleaning extractions.

On the other hand, extractions with ethanol allow the removal of the organic pollutants that are present in butts. Thus, the ^1^H-NMR spectrum of butts without extraction (Figure 7a) shows peaks at 2 and 4 ppm, corresponding to protons of triacetin used as adhesive in butts. Moreover, there are two peaks at 8.5 ppm that are due to the aromatic protons of nicotine. These peaks are not found in the cleaned butts spectrum (Figure 7b).

**Figure 8:**
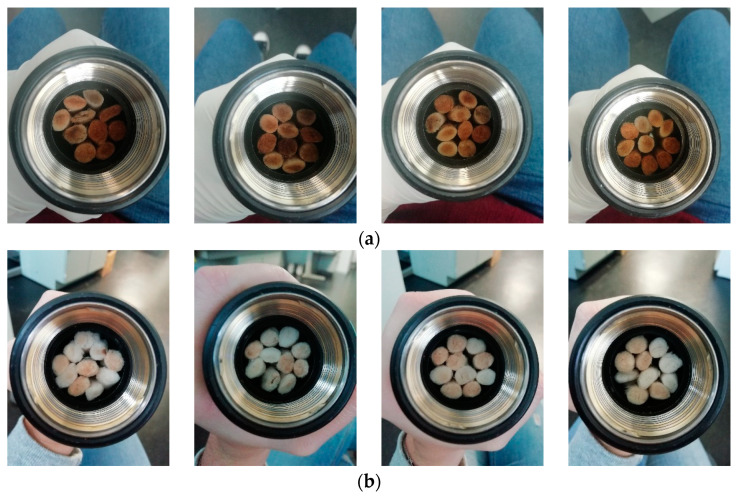
Effect of chemical cleaning on the appearance of the cigarette (**a**) before and (**b**) after cleaning.

An important challenge regarding the cleaning procedure is that, importantly, it might not alter the good acoustical properties of the samples observed before cleaning. To analyze this aspect, 4 samples of 10 used cigarette filters were used. First, they were dried and measured. Then, the cleaning procedure was applied to the different used cigarette filter groups, and, finally, the cleaned filters were dried again. In Figure 8, photographs of the four samples, before and after the application of the cleaning procedure, are shown. As can be seen, there is a clear improvement in the appearance of the cigarette filters.

**Figure 9:**
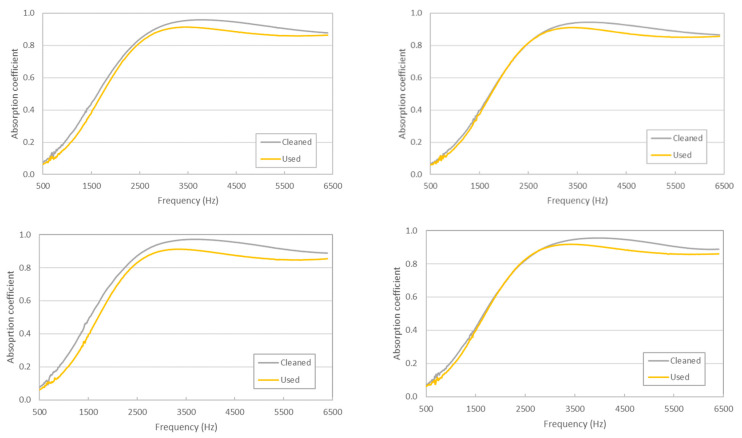
Effect of chemical cleaning in the absorption coefficient values.

In Figure 9, values of the absorption coefficients before and after chemical cleaning are shown.

As can be seen in Figure 9, samples after the cleaning procedure present higher absorption coefficients than those of the non-cleaned samples. This is a surprising result when considering that, after the cleaning procedure, a decrease in the weight of the samples (15 ± 3% in average) can be observed. A possible explanation for this absorption increase could be that, after the cleaning procedure, the filters are less rigid and occupy a larger volume of the impedance tube holder and that there is less airflow resistance (unfortunately, in a tube holder of this size, it is not possible to measure airflow resistivity), meaning that waves can more effectively penetrate the material. Moreover, there can also be an increase in the absorption surface of the filters’ fibers after the cleaning procedure.

**Figure 10:**
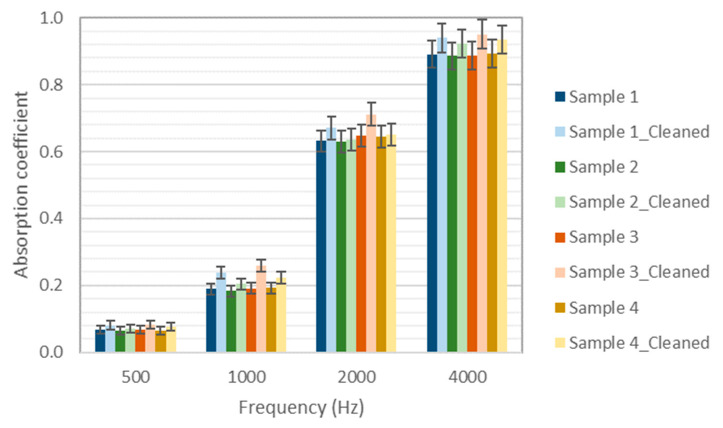
Effect of chemical cleaning on the absorption coefficient values (octave bands).

Increases in absorption are also observed in octave bands (see Figure 10) and in the average values (in the 500–6400 Hz range) of 0.45, 0.44, 0.45, and 0.45 for the non-cleaned samples and 0.48, 0.46, 0.50, and 0.47 for the cleaned ones.

## 4. Conclusions

From the results and analysis, the following conclusions can be drawn:

- The moisture content of the samples has an influence on both their weight and acoustical properties (in the values of the absorption coefficient) due to the increase in the difficulty of the sound wave to penetrate the material. A drying period of three hours at 80 °C or 105 °C seems sufficient to complete the drying of the samples.
- A chemical cleaning procedure based on diluted aqueous solutions and ethanol was proposed to eliminate the major metal ions present in the samples, as well as organic pollutants. With the proposed procedure, an improvement in the samples’ appearance was observed. The acoustical behavior of the samples improved following the chemical procedure.
- An improvement in the absorption capacity of the cigarette butts was observed when the total fiber content of the samples is more homogeneously distributed in the sample (not associated to each filter).
- With the proposed previous conditioning of used cigarette butts (cleaning and drying them), the new prepared samples keep high absorption coefficient values for medium and high frequencies, showing their potential application as acoustic absorbers. In any case, further studies related to the different influences on absorption—for instance, of density or thickness—and the possibility of the establishment of a standard method for preparing large samples would be desirable.

Finally, it is worth mentioning that the main goal of this chemical cleaning study was to eliminate pollutants from cigarette butts in order to research their acoustical behavior once they are clean. However, bearing in mind the 3R’s—recover, recycle, reuse—further research is needed (and is under development in our laboratories) addressing the elimination of the extracted metals and organic compounds, from either the aqueous or organic solvents, in order to recover them for additional use.

## Figures and Tables

**Table 1:** Variation in weight of samples after initial drying and after remaining in a saturated environment for 50 days.

Sample	Initial Weight (g)	Dried Weight (g)	Weight after 50 Days in a Saturated Environment (g)	Increase in Weight (%)
UBF1	21.4	19.4	22.9	17.9
UBF2	20.9	18.9	22.4	18.8
NUBF1	22.2	20.2	24.0	18.9
NUBF2	21.8	19.6	22.7	15.5
Mineral Wool 1	4.1	4.0	4.8	21.0
Mineral Wool 2	5.0	4.9	5.6	13.4

**Table 2:** Variation of weight of samples (%) after first drying at 105 °C and 80 °C.

Hours of Drying	Dried at 105 °C			Dried at 80 °C		
	UBF1	NUBF1	Mineral Wool 1	UBF2	NUBF2	Mineral Wool 2
3	−15.5	−15.9	−16.6	−17.69	−13.68	−10.47
24	−0.0	−0.2	0.0	0.0	−0.1	−0.2
48	−0.3	−0.2	0.0	0.0	0.0	0.0
72	−0.2	0.0	0.0	−0.2	−0.1	−0.2
144	−0.2	−0.1	0.0	0.0	0.0	0.0

**Table 3:** Concentrations of metal ions obtained by dissolution tests on cigarette butts.

Metal Ion	Dissolution Test	
	H_2_SO_4_ aq	NaCl aq
Al (μg/L)	573 ± 1.0	35 ± 5
Mn (μg/L)	26.8 ± 0.4	173 ± 0.8
Fe (μg/L)	22.9 ± 1.1	26 ± 3
Cu (μg/L)	<10	<10
Zn (μg/L)	54.2 ± 1.1	42.7 ± 1.1
Sr (μg/L)	29.5 ± 0.4	2008 ± 1
Cd (μg/L)	<10	<10
Ba (μg/L)	117.3 ± 0.8	657 ± 1.0
Pb (μg/L)	<10	11.3 ± 1.4

## Data Availability

Data sharing not applicable. No new data were created or analyzed in this study. Data sharing is not applicable to this article.
